# Novel spatiotemporal analysis of gait changes in body weight supported treadmill trained rats following cervical spinal cord injury

**DOI:** 10.1186/s12984-017-0308-0

**Published:** 2017-09-13

**Authors:** Nathan D. Neckel

**Affiliations:** Department of Neuroscience, Georgetown University, 3970 Reservoir Rd, Washington, DC, NW 20007 USA

## Abstract

**Background:**

Common gait measures such as stride length, cycle time, and step height are not independent variables, but different aspects of the same multidimensional step. This complicates comparisons between experimental groups. Here we present a novel multidimensional gait analysis method and use this method to assess the ability of body weight supported treadmill training (BWSTT) to improve rodent stepping after spinal cord injury (SCI).

**Methods:**

In lieu of reducing a step to a collection of gait measures and comparing the means of several of these, we developed a multidimensional analysis technique that compares the step as a whole. While in a passive robotic gait training device, the pre-injury hindlimb stepping of 108 rats was recorded while they walked in a quadrupedal posture at 8 cm/s. Following a C4/5 over-hemisection spinal cord injury the weekly changes in stepping were tracked for 17 untrained and 10 BWSTT animals for 7 weeks. The performance of trained rats was recorded during training with BWS, as well as at the end of the training week without BWS. An additional six uninjured rats were trained for 5 weeks.

**Results:**

Our novel multidimensional analysis shows that stepping is asymmetrically altered 1 week after SCI. The differences in stepping change over the following weeks, with the less impaired left hindlimb deviating further away from pre-injury than the more impaired right hindlimb. Uninjured rats do not significantly alter their stepping over 5 weeks. BWSTT improves the stepping of the right hindlimb, but only when the BWS is active. If the BWS is not present, the performance of trained animals is worse than untrained rats. The left hindlimb performance of BWSTT rats is worse than untrained rats, during both training sessions and weekly assessments.

**Conclusions:**

We feel that our novel multidimensional analysis is a more appropriate method to address the inter-dependencies of gait measures. Untrained rats exhibit both initial impairments as well as the development of compensatory techniques. BWSTT does not improve this spontaneous recovery, but exacerbates it, particularly in the less impaired left hindlimb.

## Background

Gait training to induce the restoration of locomotion following neurological injury has endured a bumpy translation from the pioneering bench work in spinal cats to today’s sophisticated clinical devices [1, 2]. In the quest to refine and maximize this treatment technique there currently appears to be a shift from the bedside back to the bench. Body weight support trolley systems used in recent rodent experiments [3, 4] are analogous to the established clinical ZeroG device [5]. Robotic gait training devices made their way into animal models [6] years after the development of the Lokomat [7]. And it is not just the training devices that are shifting from the clinic back to the lab, but the techniques used to measure their effectiveness. EMG, motion tracking, and ground reaction forces have been commonplace in the field of human biomechanics for years but are only recently being adopted in rodent studies.

Our own work strives to improve body weight supported treadmill training (BWSTT) by employing the use of rodent models of spinal cord injury (SCI). And like a handful of our peers we believe that training rats in a quadrupedal posture after an incomplete injury will provide more clinically relevant information [8–10]. However, unlike our peers we don’t believe that changes in stepping can be accurately expressed as a collection of mean values. Traditionally a step is separated into common measures such as step height, stride length, and cycle time, and means of these values are then compared between groups. But a step is a highly coordinated, extremely nuanced task where the spatiotemporal aspects are physically linked together and not separate entities. We set out to continue the trend of incorporating more biomechanics into rodent SCI research and developed a novel multidimensional gait analysis method that maintains the connection of a step’s position and timing. This method was used to quantify the initial deficits in stepping, as well as the development of secondary impairments, following a cervical over-hemisection injury in rats. Likewise, we used or novel gait analysis technique to assess the effectiveness of BWSTT to improve the spontaneous recovery following injury.

## Methods

### Animals and study design

All protocols were approved by the Georgetown University Animal Care and Use Committee (#2016–1222). Adult female Sprague-Dawley rats were used, 108 for the spinal cord injury studies (appx 5 weeks old, 160-220 g range, 186 ± 12 g mean, Taconic Farms, Germantown, NY) and six for the non-injured controls (appx 7 weeks old 213-251 g range, 230 ± 16 g mean, Taconic Farms, Germantown, NY). To minimize the effects of differences in size and age we focused on week to week changes in gait and not differences between groups. All animals were housed in the Georgetown University Division of Comparative Medicine with unlimited access to food and water. Neither food deprivation nor food rewards were used as motivators. These animals are part of our ongoing robotic gait training studies, and 74 of these animals were previously reported in our overground locomotion work [11, 12]. Presented here for the first time is novel analysis of BWSTT data.

**Fig. 1:**
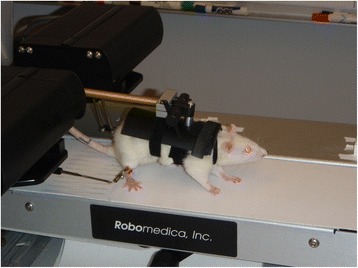
BWSTT of Rats. The Rodent Robotic Motor Performance System (RRMPS) was used to track the position of the hindlimbs (ankle cuffs) as rats walked in a quadrapedal posture on the treadmill at 8 cm/s under different levels of body weight support

The Rodent Robotic Motor Performance System (RRMPS, Robomedica, Inc., CA, Fig. 1, see [13, 14] for details) was used to passively measure the parasagittal hindlimb ankle position of rats as they walked in a natural quadrapedal support posture on a treadmill traveling at 8 cm/s under varying levels of body weight support. While this belt speed is a slow pace for healthy rats, it is challenging, but achievable, for animals 1 week after SCI. The robotic actuators of the RRMPS device only attach to the hindlimbs of the rats, allowing the forelimbs to step freely. Unfortunately this also means that we cannot record the forelimb stepping. Thus analysis of the interlimb coordination of these quadrupedal animals and the interplay between forelimb dysfunction and hindlimb performance following injury (as well as hindlimb training and forelimb recovery) is reserved for our work with unassisted overground locomotion.

Rats were pre-trained for seven non-consecutive days before pre-operative baseline recordings at 0% BWS for 2 min. Rats received a right over-hemisection injury at the C4–5 level (previously described in [15, 16]). Briefly, rats were anesthetized with 3% isoflurane, a partial C4/C5 laminectomy was done, and iridectomy scissors were used to create a lesions at C4–5. The lesion bilaterally ablates the dorsal corticospinal pathway, and unilaterally ablates the contralateral rubrospinal pathway resulting in profound asymmetric impairments, with the right side more impaired than the left, and the forelimbs more impaired than the hind. However, 1 week after injury rats are able to independently transverse the glass walkway of the CatWalk gait analysis device. Thus this model is ideally suited to investigate the ability of robotic gait training to improve overground locomotion. At the end of the study all lesion sites were reconstructed from serial cresyl violet sections or MRI images and only the 92 animals with appropriate injuries were included in post-injury analysis.

Seven days after injury all rats were re-assessed on the RRMPS at 0% BWS for 2 min. Beginning on post injury day 8 and continuing through post injury day 31, a subset of 10 animals were trained Monday through Thursday on the RRMPS at decreasing levels of BWS and increasing lengths of training time (data from the Wednesday session hereafter referred to as weeks 1.5 through 4.5). Weekly 2 min assessment at 0%BWS were conducted on Fridays, starting on post injury day 11 and ending on post-injury day 46 (hereafter referred to as weeks 2 through 7). Week 1 training was 75% BWS for 5 min, week 2 began at 75% BWS but was reduced to 40% BWS by weeks end, for 10 min. Training weeks 3 and 4 were 40% BWS for 15 total minutes. To reduce animal fatigue these sessions were split in to an 8 min session, a 60 min break, and a 7 min session. All data was recorded from the first 2 min of that day’s session.

The RRMPS device provides BWS by precisely rotating a cantilever beam which in turn raises the rats’ custom fitted vest. We aligned the animals within the device to ensure that a quadrupedal posture was maintained. Even at 75% BWS, when an animal went limp and refused to participate, the entire chest and belly made contact with the treadmill belt and all four limbs dragged behind the animal. This assured us that even though we are unable to measure the specific weight distribution to the four paws, we do not believe the RRMPS device was providing significantly unequal loading between the forelimbs and hindlimbs.

A different subset of 17 injured animals were not trained but only assessed weekly on Fridays on the RRMPS at 0% BWS for 2 min. The six uninjured controls were trained at 0%BWS for 2 min, three times a week (Monday, Wednesday, Friday), for five consecutive weeks. Uninjured controls were used instead of true sham injuries to investigate any training effects on healthy, intact animals. These weeks are analogous to injured weeks −1 through 4 (as seen in Table 1).

**Table 1:**
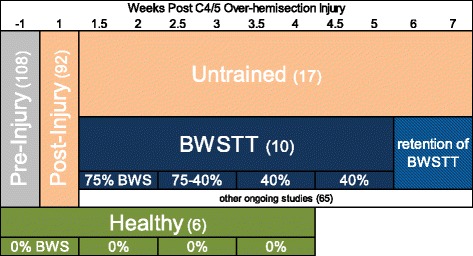
Experimental timeline

### Spatiotemporal data extraction

Individual steps were extracted from the two minute recordings by using the following algorithm. First, the vertical and horizontal trajectories of each hindlimb that were recorded at 500 Hz were loaded into custom software (MATLAB, Mathworks, Natick MA), low-pass filtered (forward and backward to prevent phase lag) with a 4th-order Butterworth filter with 9 Hz cutoff frequency, and screened to find local maximums and minimums. Sequences of min-max-min in the horizontal trajectory (*x*) were isolated as possible forward and back movements of the limb. The vertical trace (*y*) was then checked over the same time interval to see if there was a corresponding min-max-min as a possible up and down movement of the limb. When in agreement, these up and forward trajectories in the unfiltered data were taken as successive toe offs (TO). The majority of rodent robotic gait training studies place the animal in a bipedal posture where forward motion is considered swing phase, backward motion is stance phase, and initial contact (IC) is the transition [13]. However in the quadrapedal posture tested here a more robust definition of initial contact is needed. A region of the filtered vertical trajectory after the horizontal velocity changed from positive to negative was screened for rapid changes from negative velocity, to positive velocity. The earliest instance of this indicated a reactive “bump” when the paw contacts the treadmill belt. The unfiltered vertical minimum of this “bump” was taken as the initial contact with the treadmill and the beginning of stance phase. Following injury rats would often let the more impaired right limb drag behind before initiating bouts of sporadic stepping. Step cycles were therefore analyzed from TO to TO to capture this variation in swing phase.

### Gait analysis in robotic trainier

**Fig. 2:**
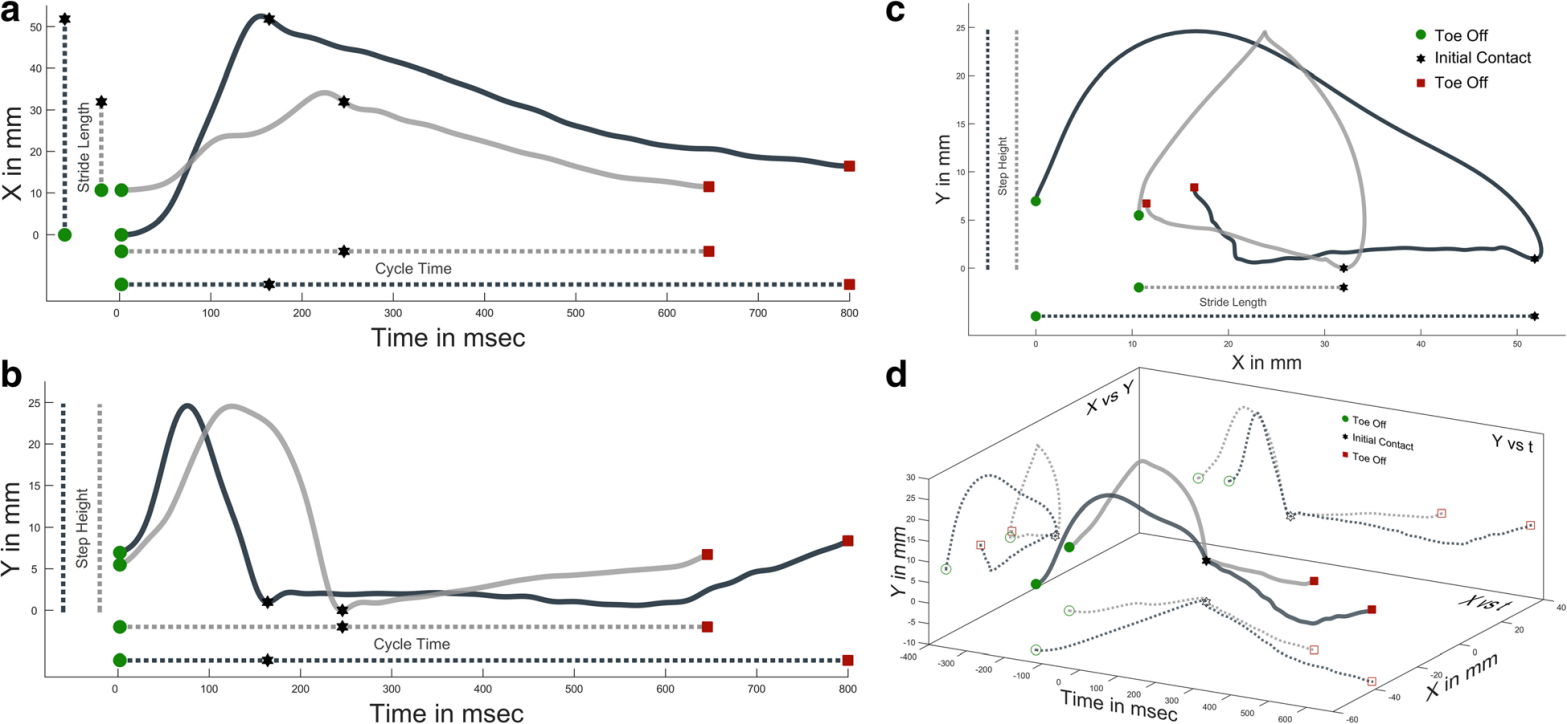
Multidimensional Differences Between Two Steps. Two non-consecutive steps from the same training session of a pre-injury rat. As a step progresses from toe off (green circle), to initial contact (black star), to subsequent toe off (red square) the *x,y,t* coordinates are recorded. Traditional 2D representations of this data are found in plots **a-c** with classic gait measures shown as 1D projections of the 2D data. **d** shows the 2D plots of **a-c** as projections of a 3D representation of the steps, with initial contact at the origin

Initially we set out to analyze classic gait measures such as step height, stride length, and cycle time. But we came to realize that comparing means of such measure did not adequately represent the differences in steps. Fig. 2 shows spatiotemporal plots of two non-consecutive steps from the same training session of a pre-injury rat, along with their projections into classic gait measures. The difference in stride length can be seen in Fig. 2a and c, and the difference in cycle time in 2a and b. There is no difference in step height as measured by peak height (*y*_*y peak*_), but Fig. 2b and c show that the step heights are different in terms of time of peak height (*t*_*y peak*_) and anterior/posterior position of peak height (*x*_*y peak*_). When comparing pre/post injury, or pre/post treatment performance, we were unsure of how to analyze this difference - are step heights really the same if they happen at different times in the gait cycle, or in different anterior/posterior positions? This led us to appreciate that classic gait measures such as step height (*y*_*y peak*_) are not independent from their spatiotemporal components in the *x* and *t* domains. And while MANOVA and other corrections address the dependency between *x,y,t*_*peak*_, we could not escape the fact that many gait measures are not just dependent variables, but physically linked together. Thus we developed a technique to embrace this link instead of separating it out into multiple components.

### 3D modeling of stepping

**Fig. 3:**
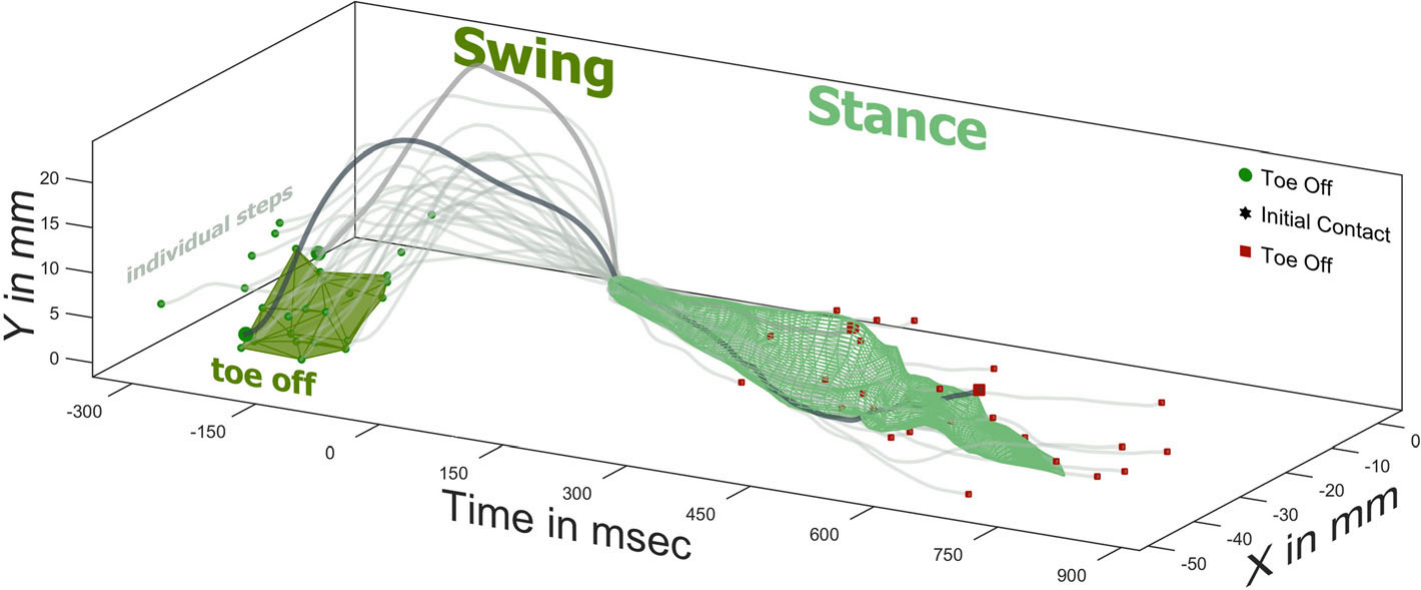
Grouping of Steps in 3D. The two steps from Fig. 2 are plotted with the remaining steps from the same session of a pre-injury rat. For each percent of the gait cycle a cluster of the 68.27% of points that are nearest each other are fitted with a boundary volume. The boundary volume for 1%, or toe off, is represented in dark green. For visual inspection, multiple boundary volumes are merged and fitted with a smoothed mesh. The stance phase location in *x,y,t* space of 68.27% of steps are found within the light green mesh

**Fig. 4:**
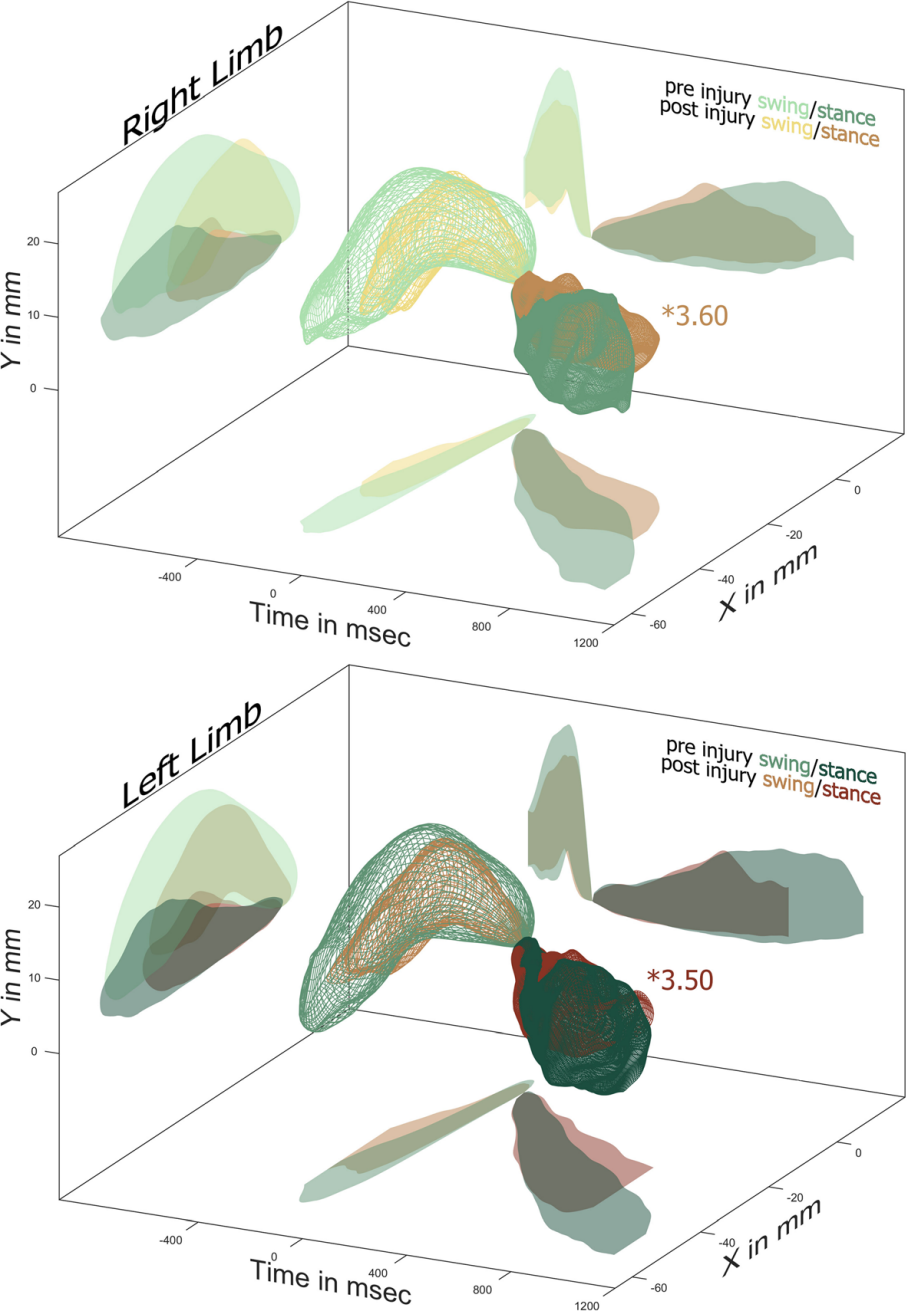
SCI Impairs Stepping. One week after a C4/5 right over-hemisection injury the stepping of both hindlimbs are significantly different from pre-injury. The 3D meshes are shown along with their projections onto common 2D planes. The most likely source of the differences following injury is the reduction of cycle time in the left limb and the anterior shift in stance of the right limb

If step height is just the 1D projection of the 2D *y*,*t* plot, then it stands to reason that the *y*,*t* plot itself is just a 2D projection of a 3D *x*,*y*,*t* plot (Fig. 2d). This 3D representation of a step is the basis of our technique. To start we cubically splined each step to 100 points (1point = 1% gait cycle) and plotted the steps in 3D *x,y,t* space. In our set-up the pelvis is not rigidly fixed or tracked so to combine steps from like groups it was necessary to shift the data out of the device coordinates and into the step coordinates by making initial contact the origin. This has the added benefit of making swing phase negative time, and stance phase positive. The time domain was compressed by a factor of 0.075 so that its range was between the range of *x* and *y*, making error similar in all dimensions. Fig. 3 illustrates the process for the single pre-injury session of the rat from Fig. 2, but the process is the same for any group of steps. When comparing experimental groups of rats we only took a maximum of 20 steps per rat. And the 20 steps were not randomly selected, but the 20 steps that were most similar to each other. For each percent gait cycle a custom cluster analysis was performed to find the single continuous cluster of 68.27% of points that were nearest each other. This cluster of points was then fitted with a 3D triangulated volume (MATLAB boundary function, .5 shrink factor) which served as our model, and the remaining 31.73% of points outside the model had residual distances to the model (face, edge, or point). A model with a larger volume would naturally contain more points and decrease the residuals, so our error term was weighted. We determined error to be the product of the sum of residuals, the volume of the model, and the ratio of number of points outside to inside the model. This results in each percent of gait cycle having its own unique model and error. To determine if two groups of steps were different we would measure the change in error if the models were switched e.g. the error of data A in model A plus the error of data B in model B should be less than A in B plus B in A [as in 11]. This change in error was then summed across the entire gait cycle. If it is assumed that the 108 pre-injured rats take symmetric steps then the error increased by switching the right and left limb models would give us the limits of our technique. We determined significant difference between experimental groups as anything greater than twice this healthy right/left error limit. Differences in performance in the robotic gait trainer are expressed as this increase in relative error (RE), with anything greater than two being significant [11]. To visualize the entire gait cycle as in Figs. 3, 4, and 6 the volumes from each percent gait cycle were combined and fitted with a smoothed mesh (smoothingspline, 0.8).

## Results

Our novel multi-dimensional analysis strives to analyze all spatiotemporal aspects of gait equally and simultaneously, with the trade-off being lack of specificity. So while we cannot make statements like “there is no significant difference between pre/post injury swing time”, we also do not encounter the conundrum of similar swing times following different swing paths. When plotted in 3D with 2D projections visual inspection can be used to infer the possible sources of the significant differences found in overall spatiotemporal relative error (RE).

### SCI impairs stepping

One week following a right C4/5 over-hemisection injury the stepping patterns of both hindlimbs are significantly different from pre-injury values. Inspection of the 3D plots in Fig. 4 reveals that following injury the left hindlimb has a shorter cycle time with the reduction coming from stance phase. The post injury left hindlimb has a shorter stride length but also moves further anterior in early swing and not as far posterior in mid to late stance. Even though peak step height occurs at the same time before initial contact, there is a reduction in post injury step height with a corresponding anterior shift. In general the post injury left hindlimb swing phase lasts just as long, and travels through a similar, albeit smaller, triangular path. One week following injury the impairments found in the right hindlimb are similar to those observed in the left. This is reflected in the very similar relative errors when compared to pre-injury (RE 3.50 for the left, RE 3.60 for the right) and low, but significant, level of asymmetry (RE 2.67).

**Fig. 5:**
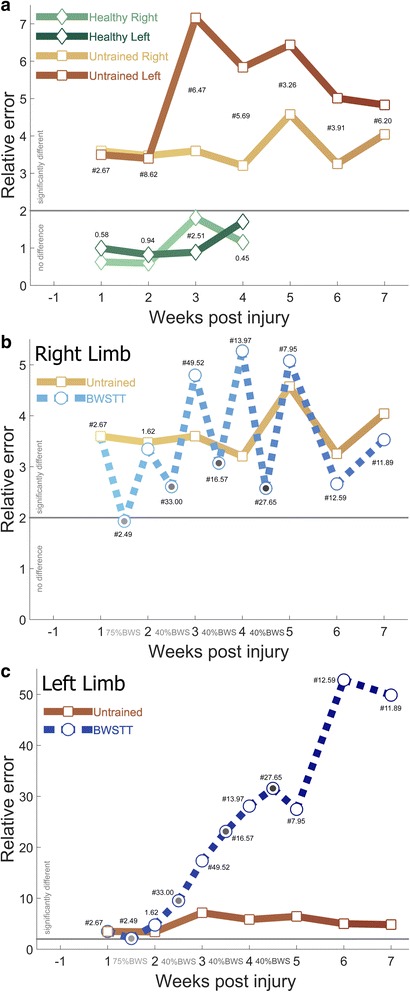
Stepping Impairments Change Over Time, Are Not Improved With BWSTT. **a** Healthy animals do not alter their stepping over 5 weeks of training as measured by relative error no greater than twice the difference between right and left limbs from week −1. One week following injury untrained injured animals are significantly different, with the less impaired left hindlimb spontaneously deviating further away as the weeks progress. This is interpreted as a development of compensatory techniques. **b** BWSTT improves right hindlimb stepping during the active training sessions (gray dots between weeks) but induces a deleterious after-effect when the BWS is removed for weekly assessments. This effect washes out the 2 weeks after the cessation of training. **c** BWSTT does not improve left hindlimb stepping, with little or no evidence of an after-effect, and no evidence of a washout following the cessation of testing. Levels of limb asymmetry for that week are found near each marker, with # indicating significance

As the weeks progress the 17 untrained injured animals spontaneously change and adapt their stepping patterns (Fig. 5a). The more impaired right limb is significantly different from pre injury throughout the 7 weeks of testing and maintains a relatively steady level of error with week 5 max of RE 4.57, and finishing the experiment at RE 4.04. The less impaired left limb is significantly different from pre injury throughout the 7 weeks of testing but this difference is not constant. By 3 weeks post injury the left hindlimb further deviates away from pre injury levels to a peak difference of RE 7.16 before gradually reducing this difference to RE 4.83 by week 7. This greater amount of change in the less impaired hindlimb several weeks after injury is consistent with our previous analysis of overground locomotion, and indicative of the adoption of compensatory techniques. The untrained injured animals exhibit significant levels of asymmetry throughout the study, but with no discernable pattern.

### Gait training does not alter healthy stepping

Six uninjured controls were trained in the RRMPS under 0% BWS for 2 min three times a week (Monday, Wednesday, Friday) for 5 consecutive weeks. The stepping of healthy rats never deviated from their initial week 1 performance. There was a modest asymmetry in the 4th week (RE 2.51), but it did not persist into the 5th week.

### Gait training alters stepping following SCI

Starting on post injury day 8, ten rats entered the BWSTT paradigm. For four consecutive weeks the rats were trained daily (Mon-Thurs) with decreasing levels of BWS for increasingly longer sessions (week 1–75%/5 min, week 2–40%/10 min, weeks 3&4–40%/15 min). The first 2 min of the Wednesday training sessions were recorded and each Friday a 2 min assessment was recorded with no BWS. Fig. 5b illustrates the effects of BWSTT on the more impaired right hindlimb while Fig. 5c shows the effects on left hindlimb. The right hindlimb shows a trend of BWS having a positive effect on stepping, with less error than the untrained injured animals during the Wednesday sessions when the BWS was active. This reduction in error was particularly pronounced in week 1.5 when 75% BWS enabled the right hindlimb stepping to be no different from pre-injury (RE 1.93). Unfortunately this improvement in stepping during BWSTT was countered with a worsening in stepping when the BWS was off during the Friday assessments. From post injury weeks 3–5 BWSTT rats had worse stepping than untrained injured rats. BWSTT was stopped after 4 weeks but weekly Friday assessments continued for two more weeks to gauge how much training was retained. The right hindlimb stepping of the rats that were in the BWSTT paradigm did have less relative error than untrained injured rats, but were still significantly different from pre-injury.

The less impaired left hindlimb shows a trend of BWSTT having a negative effect on stepping. The left hindlimb was the most similar to uninjured stepping in week 1.5, when the robotic gait trainer was applying 75%BWS (RE 2.12). From week 2 onward BWSTT animals performed much worse than untrained injured animals. This was irrespective of if BWS was being applied or not.

With regards to asymmetry, there was no difference between right/left stepping 2 weeks after injury (RE 1.62). This was after 1 week of training at 75%BWS. Three weeks after injury the asymmetry peaked at RE 49.52. This was after 1 week of training at 40%BWS. From the peak of week 3 there was an uneven reduction of asymmetry to RE 6.20 by week 7.

**Fig. 6:**
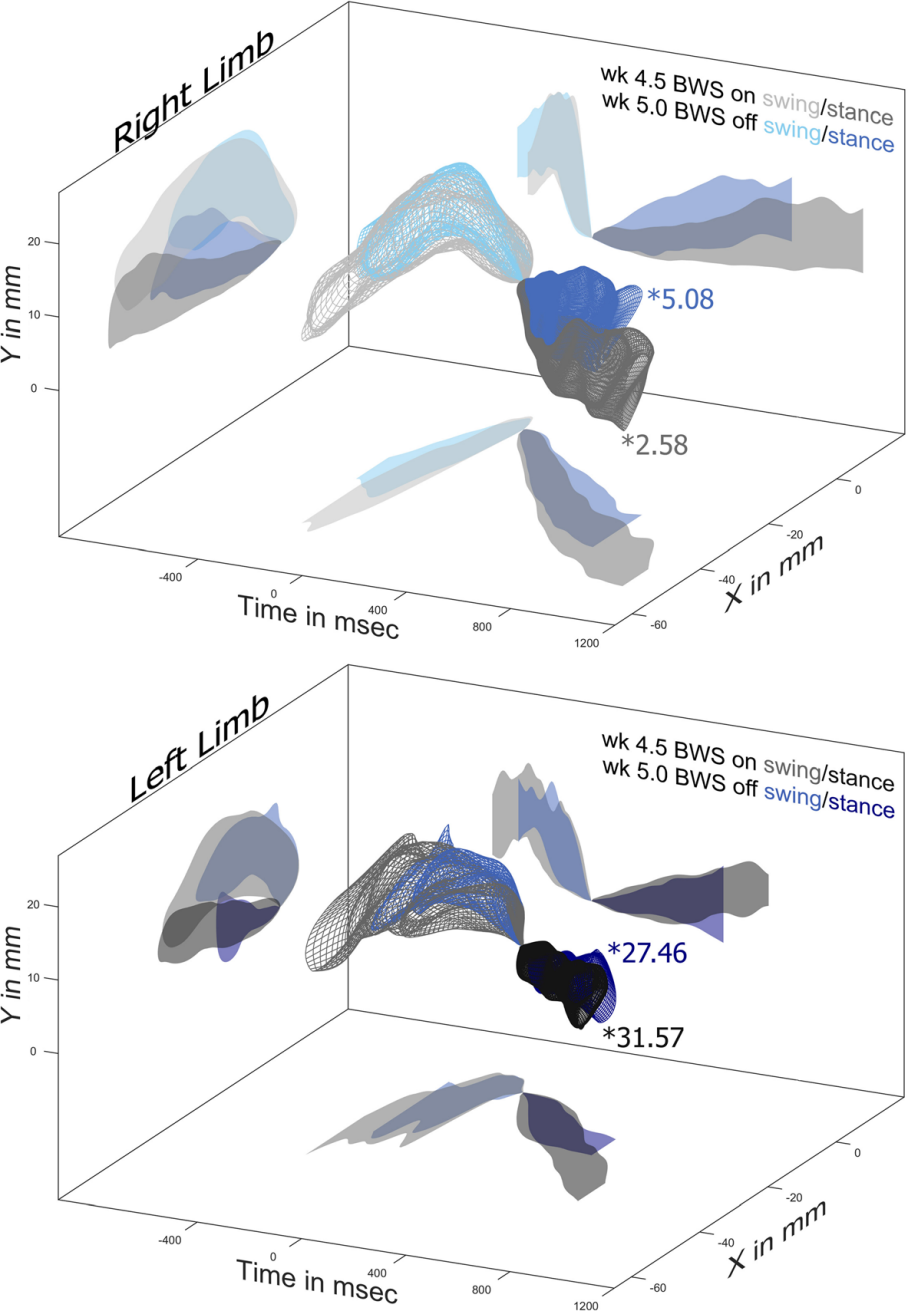
Effects of 40% BWS on Hindlimb Stepping. During the week 4.5 training session, with 40%BWS on, the right hindlimb is more like pre-injury (*2.58) than at week 5 with BWS off (*5.08). The most likely source of this difference is the longer stance time and longer stride length with 40% BWS. The left hindlimb is very different from pre-injury, with (*31.57) or without (*27.46) BWS

A more detail view of the differences in stepping brought about by 4 weeks of BWSTT can be found in Fig. 6. Midway through the training week, with 40%BWS the right hindlimb is only slightly different from pre-injury stepping (RE 2.58). At the end of the training week when the animals are assessed without the aid of BWS the right hindlimb performs worse (RE 5.08). Visual inspection reveals that the most likely cause of this increase in error comes from the shorter stride length and shorter stance time. The left hindlimb behaves similarly, with 40%BWS inducing a slightly longer stride length and longer stance time. However, the relative errors shift in the opposite direction, with 40% BWS increasing error from RE 27.46 to RE 31.57. The left hindlimb stepping is so different from pre-injury that the increases in stride length and stance time do little to improve the error. Visual inspection of Fig. 6 indicates that the lower error in week 5 could be from the lower height of late stance as well as the higher and more anterior position of late swing.

## Discussion

Through the course of our studies we have come to appreciate that gait is a complex activity, so techniques used to measure changes in gait need to be sophisticated enough to capture this complexity. Common gait measures like stride length, cycle time, and step height are not independent discrete units, but in fact simpler aspects of a much larger connected system. That is why we have shied away from reducing everything to a mean and running multiple ANOVA tests and sought to develop a technique to quantify stepping as a whole. Presented here is a method to express the multidimensionality of a step, group like steps together, and compare the difference in stepping between experimental groups. With this technique we are able to show the overall spatiotemporal gait impairments following a right C4/5 over-hemisection injury in rats. Additionally, this novel gait analysis technique enables us to examine the effectiveness of body weight supported treadmill training (BWSTT).

While there already exist tried and true statistical methods to analyze multiple dependent variables (MANOVA, PCA) we were not comfortable using these for gait analysis. We feel that there is an important difference between correcting for any interactions between variables and acknowledging that the variables are physically linked together. That is why we did not want to separate out step height, time of peak height, and anterior/posterior position of peak height only to have to go back and correct for their dependencies. We wanted to keep their dependencies intact. In fact, their interactions are the gait differences that we want to compare between groups.

The RRMPS device reports the endpoint position of the robotic actuator in the parasagittal plane over time. So plotting these three variables (*x,y,t*) in 3D space is rather straightforward. But locomotion is hardly just the time/location of an ankle cuff. It would have been wonderful to also track the *x,y,t* location of the joints. There is nothing preventing our proposed technique from analyzing multiple points in parallel and simply summing the total relative error. This could then transform the routine stick figure drawing found in many animal model gait studies into a powerful quantitative tool. However, the same trade off between specificity and multiple dependent variables exists. So while adding joint markers would give a more complete picture of changes in gait, we would still not be able to make specific claims such as “knee position at initial contact is significantly more anterior.” Adding joint markers also enables more dimensions to be analyzed. For instance, the knee can be represented in three dimensions of space (*x,y,z*), one of time (*t*), and joint angle (θ). While we believe it can be done, five dimensional analysis of gait is outside the scope of this study.

Validation of our novel technique is found by taking a closer look at the performance of the healthy animals. Figs. 2 and 3 show the expected variation of steps found within the healthy population. No two steps are going to be the same; no two rats are going to be the same. However, when all the steps from all the rats are pooled together a general model of behavior forms. Over the 5 weeks of training the healthy animals do not significantly alter this model of their stepping. This is not surprising, as we have found similar results with healthy young adults in the Lokomat robotic gait orthosis [17]. If our novel technique lacked consistency in evaluating the healthy animals it would certainly cast doubts on our process.

Whereas the healthy animals are consistent, the untrained injured animals are anything but. One week following a C4/5 over-hemisection injury the hindlimb stepping is both significantly asymmetric and significantly different from pre-injury. Two weeks after injury the level of asymmetry increases while the differences from pre-injury stay similar. While not the prettiest of data, it can best be explained by envisioning the two groups shifting in opposite directions, so they are more different from each other than they are from the previous weeks model (which is somewhere between them). From 3 weeks after injury onward, we see a more convincing result with a high level of asymmetry and a large difference from pre-injury. The fact that the left hindlimb, with less interruption of supraspinal drive (C4/5 over-hemisection leaves rubriospinal and reticulospinal tracts intact on the left side) is the limb to exhibit a greater deviation from pre-injury, and not until several weeks after injury, is of no surprise. In our previous analysis of overground locomotion of a subset of these animals we concluded that the less impaired limbs, with their greater level of control and range of motion, will adopt abnormal gait patterns in an effort to enable the more impaired limbs to complete an effective step [11, 12]. And because this change in left limb stepping does not happen until several weeks after injury, we believe this is further evidence of the development of a compensatory technique in the left hindlimb following a cervical over-hemisection injury. This is also consistent with previous studies in lateral thoracic hemisected rats where differences in means of discrete measures of overground locomotion were found starting 3 months post injury [18].

In an effort to both restore the locomotor impairments, and limit the development of compensatory techniques following SCI, a subset of rats entered a body weight supported treadmill training (BWSTT) protocol. We followed the standard clinical practice (which itself is based on spinal cat experiments [9]) of lowering the level of BWS and increasing the duration of training as the weeks progressed to maintain a challenging, yet achievable, training experience. However, just because performance may be good during training, does not mean training is good for performance. That is why we conducted weekly assessments without BWS, even for 2 weeks after the cessation of training. It should be noted that these are not “catch trails” where the BWS is suddenly turned off, but independent recording sessions at least 24 h after the most recent bout of BWSTT. The more impaired right hindlimb performs very differently during training with the BWS on than during assessments with the BWS off. With BWS on, the right limb of injured animals performed better than untrained injured animals. And when this BWS was at 75% the right limb is no different from pre-injury, even though it is a mere 9 days after injury. However, when the BWS was off the animals performed worse than when it was on. And for the final 3 weeks of training (40% BWS), the BWSTT animals performed worse than untrained injured rats during the Friday assessments. It is as if BWSTT at 40% has a negative after-effect. This indicates that training at 75% BWS throughout the course of the experiment may have been a more optimal paradigm. For the 2 weeks after the cessation of training the right hindlimb is very similar to untrained injured animals, if not slightly better. We speculate that this slight improvement would wash out had the experiment been carried out further.

Interestingly the left hindlimb does not exhibit any consistent difference between training session or assessment session, and simply drifts away from pre-injury levels as the weeks progress. The untrained injured animals do not perform this way, so there is a deleterious effect of our BWSTT (except for 1 week of 75% BWSTT). It may be that BWSTT enables the left limb to take on more drastic compensatory techniques, especially the shorter cycle time seen in Fig. 6. Again, perhaps maintaining 75% BWS throughout the course of the experiment would have been more beneficial to the left hindlimb as well.

We would like the results presented here not to be seen as a strike against BWSTT, but more of an illustration of how training effects performance, often in unintended ways. The RRMPS device we used to provide BWS is a fully programmable robotic gait training device. And as we move forward with robot assisted gait training (RAGT) it is our intention to build off of the after effect findings presented here. Good performance during training is not the goal. Training that induces better performance after the sessions are complete is the main aim of our future studies.

## Conclusions

Traditional analysis of rodent stepping separates the spatiotemporal data into many different measures such as step height, stride length and cycle time. Means of these measures are compared and presumably, hopefully, the dependencies of these many measures are corrected for post-hoc. Presented here is a novel gait analysis technique that maintains the dependencies of spatiotemporal gait data by treating the step as a whole. One week following a right C4/5 over-hemisection injury our novel technique revealed that both hindlimbs are significantly different than pre-injury. But as the weeks progressed, the less impaired right hindlimb deviated further away from pre-injury stepping. In an effort to improve upon this spontaneous recovery a group of animals entered a BWSTT paradigm. The application of BWSTT improved the stepping of the more impaired right hindlimb, but also induced a deleterious after-effect when the body weight support was not present. The less impaired left hindlimb of BWSTT rats shifted further away from pre-injury than untrained injured rats. It appears that BWSTT has the ability to help the more impaired limb to take better steps while also assisting the less impaired limb adopt more drastic compensatory techniques.

## Abbreviations

BWSTT: Body weight supported treadmill training
C4/5: The level of the spinal cord between the 4th and 5th cervical roots
RAGT: Robot assisted gait training
RE: Relative error
RRMPS: Rodent Robotic Motor Performance System (Robomedica, Inc., CA)
SCI: Spinal cord injury

## References

[1] Barbeau H, Rossignol S (1987). Recovery of locomotion after chronic spinalization in the adult cat. Brain Res.

[2] de Leon R, Dy C (2017). What did we learn from the animal studies of body weight supported treadmill training and where do we go from here?. J Neurotrauma.

[3] van den Brand R (2012). Restoring voluntary control of locomotion after paralyzing spinal cord injury. Science.

[4] Hamlin M (2015). A novel device for studying weight supported, quadrupedal overground locomotion in spinal cord injured rats. J Neurosci Methods.

[5] Nichols D (2011). ZeroG: overground gait and balance training system. J Rehabil Res Dev.

[6] Nessler JA (2005). A robotic device for studying rodent locomotion after spinal cord injury. IEEE Transactions on neural systems and rehabilitation engineering.

[7] Jezernik S (1999). Robotic orthosis lokomat: its use in the rehabilitation of locomotion and in the development of the biology-based neural controller.

[8] Shah PK, et al. Use of quadrupedal step training to re-engage spinal interneuronal networks and improve locomotor function after spinal cord injury. Brain. 2013:awt265. 10.1093/brain/awt265 PMC3808689 24103912

[9] Ward PJ (2014). Novel multi-system functional gains via task specific training in spinal cord injured male rats. J Neurotrauma.

[10] Ward PJ, et al. Training-Induced Functional Gains following SCI. Neural Plasticity. 2016;2016 10.1155/2016/4307694 PMC4926009 27403345

[11] Neckel ND. Methods to quantify the velocity dependence of common gait measurements from automated rodent gait analysis devices. J Neurosci Methods. 2015;253:244–53. 10.1016/j.jneumeth.2015.06.017 PMC4560675 26129742

[12] Neckel ND, Dai H, Bregman BS (2013). Quantifying changes following spinal cord injury with velocity dependent locomotor measures. J Neurosci Methods.

[13] Timoszyk WK (2002). The rat lumbosacral spinal cord adapts to robotic loading applied during stance. J Neurophysiol.

[14] Nessler JA (2006). Robotic gait analysis of bipedal treadmill stepping by spinal contused rats: characterization of intrinsic recovery and comparison with BBB. J Neurotrauma.

[15] Lynskey JV (2006). Delayed intervention with transplants and neurotrophic factors supports recovery of forelimb function after cervical spinal cord injury in adult rats. J Neurotrauma.

[16] Bregman BS (1993). Recovery of function after spinal cord injury: mechanisms underlying transplant-mediated recovery of function differ after spinal cord injury in newborn and adult rats. Exp Neurol.

[17] Hidler J, Wisman W, Neckel N (2008). Kinematic trajectories while walking within the Lokomat robotic gait-orthosis. Clin Biomech.

[18] Leszczyńska AN (2015). Thoracic hemisection in rats results in initial recovery followed by a late decrement in locomotor movements, with changes in coordination correlated with serotonergic innervation of the ventral horn. PLoS One.

